# Outcome of early use of non-invasive positive pressure ventilation in patients with acute exacerbation of chronic obstructive pulmonary disease

**DOI:** 10.12669/pjms.35.6.857

**Published:** 2019

**Authors:** Nadia Ishfaq, Naheed Gul, Neelum Zaka

**Affiliations:** 1 Dr. Nadia Ishfaq, FCPS Internal Medicine, Senior Registrar, Department of Medicine. Shifa College of Medicine, Islamabad, Pakistan; 2 Dr. Naheed Gul, FCPS Internal Medicine, Associate Professor, Department of Medicine. Shifa College of Medicine, Islamabad, Pakistan; 3 Dr. Neelum Zaka, MCPS & FCPS Internal Medicine, Assistant Professor, Department of Medicine. Shifa College of Medicine, Islamabad, Pakistan

**Keywords:** Arterial blood gases, Chronic obstructive pulmonary disease, Non-invasive positive pressure ventilation, Mechanical ventilation, Mortality

## Abstract

**Objective:**

To determine the outcome of early use of non-invasive positive pressure ventilation (NIPPV) in Pakistani patients with acute exacerbation of chronic obstructive pulmonary disease.

**Methods:**

This descriptive study was conducted at Shifa International Hospital Islamabad from April 2015 to January 2017. A total of 120 patients with acute exacerbation of chronic obstructive pulmonary disease receiving NIPPV alongside standard therapy were included in the study. The patients were clinically assessed before starting on NIPPV. The parameters of respiratory rate, pH and paCO_2_ were monitored and NIPPV was given for six hours to evaluate clinical outcomes and analyze the factors predicting failure (requirement of mechanical ventilation and mortality). Frequency and percentages were calculated for qualitative variables while Mean and Standard Deviation for quantitative variables. Chi-square and t-test were used to see differences in pre and post NIPPV arterial blood gases.

**Results:**

Patients’ mean age was 58.88±10.09 years. Males were 88 (73.3%) and females were 32 (26.7%). The mean respiratory rate was 24±1.45 per minute before and 17.96±1.35 per minute after NIPPV (p < 0.00001). The mean pH before NIPPV was 7.27±0.04 and afterwards 7.38±0.02 (p < 0.00001). The mean pCO_2_ was 61.87±9.60 mm of Hg before and 57.46±6.79 mm of Hg after NIPPV (P < 0.0003). Twenty Four (20%) patients required invasive ventilation of which 19 (15.8%) patients could not survive.

**Conclusions:**

There was remarkable improvement in the arterial blood gases after NIPPV. However, the high mortality rate and significant number of COPD patients requiring mechanical ventilation necessitates further investigation into our population.

## INTRODUCTION

Chronic Obstructive Pulmonary Disease (COPD) is characterized by progressive partly reversible air flow limitation and lung hyperinflation with major extra pulmonary manifestations and co-morbid conditions.^1^ It is the major health problem and is the 5^th^ and 12^th^ leading cause of death and disability worldwide, respectively.^1^,^2^ It is the 3^rd^ leading cause of mortality in United States with an expenditure of more than 40 billion dollars.^3^ A survey including Middle East, North Africa and Pakistan reported overall prevalence of COPD to be 3.5 % with up to 2.2% of patients from Pakistan.^4^ According to WHO, the number of COPD cases worldwide will increase by three times the total number of COPD cases by the year 2020.^1^

COPD exacerbation is characterized by the deterioration of patient’s baseline symptoms including dyspnea, cough and/or sputum which usually requires change in regular medication.^1^ An acute exacerbation of COPD is the main source of admission to hospitals.^5^ A portion of these patients with frequent exacerbations may develop acute respiratory failure requiring Intensive Care Unit (ICU) admission and utilization of large ICU resources.^6^

The first line of cure in patients with Acute Respiratory Failure (ARF) is ventilation. Patients with ARF can be ventilated by either positive or negative pressure invasively or non-invasively.^7^ Mechanical ventilation can be a lifesaving procedure in patients with ARF.^5^ However, the use of artificial way leads to severe infection and carries the risk of trauma to the airways.^1^ Non-invasive ventilation is another safe approach that was developed to avoid these complications in such patients. The early use of NIPPV have shown decrease in intubation rate by 66% and mortality rate by 9%, thus minimizing ICU and hospital stay.^3^ A research study showed advantages of NIPPV in staying away from the requirement for invasive mechanical ventilation in patients presenting with ARF of various etiology.^8^ With the use of NIPPV, the mortality rate improved to 10.1% compared to mechanical ventilation (26%), while the length of stay in ICU has been reported as 7 to 13 days which is significantly better than invasive mechanical ventilation (10 to 20 days).^3^ In another large study (248 patients) conducted in India, NIPPV showed significant improvement in clinical and blood gas parameters, endotracheal intubation and mortality in certain group of patients.^7^

The use of NIPPV for improvement in arterial blood gases (ABGs) in patients with acute exacerbation of COPD has also been studied in Pakistan.^5^,^9^,^10^ The sample sizes were however quite small and data available insignificant to draw definite conclusions. Only one study has determined the effect on mechanical ventilation and mortality in 2001 with only eighteen patients.^9^ The number of patients requiring mechanical ventilation and mortality rate is not well established in our patients receiving NIPPV in combination to standard therapy for acute exacerbation of COPD. The motivation of this study was to find out the frequency of Pakistani population with acute exacerbation of COPD receiving NIPPV alongside standard treatment requiring mechanical ventilation and the overall mortality rate on a large sample size and in a tertiary care hospital with state of the art health facilities. We also investigated the effects on ABGs for comparison to previous studies in Pakistan.

## METHODS

This was a prospective hospital based descriptive study carried out in Department of Medicine at Shifa International Hospital, Islamabad. This was a longitudinal study conducted over a period of 21 months from April 2015 to January 2017. A total of 120 patients with acute exacerbation of chronic obstructive pulmonary disease were included using non-probability (consecutive) sampling technique. Sample size was calculated as 120 using WHO sample size calculator considering 95% confidence level, absolute precision 6% and anticipated population proportion p=12.5 %.

Patients with COPD exacerbation fulfilling the following criteria were included in the study: 1) All age groups 2) Both males and females 3) Dyspnoea at rest or respiratory rate > 25 breaths per minute and oxygen saturation less than 85%. 4) FEV 1<50% predicted or PEFR < 100 litter per minute. 5) ABGs showing pH<7.35, Pa Co_2_ > 45mmHg and PaO_2_ < 60 mmHg. Patients with following conditions were excluded from the study:1) Arterial blood pH < 7.20 2) Unconsciousness 3) Unable to clear their airways 4) Un-cooperative, agitated 5) Hemodynamic instability 6) A central nervous system disorder unrelated to hypercapnia associated encephalopathy or hypoxemia.

Institutional review board approval was obtained before starting data collection. (IRB 975-250-2018 dated March 31, 2018) Written informed consent was taken and a detailed history followed by rigorous clinical examination of the patients were documented. Respiratory rate and ABGs were taken before Non-invasive positive pressure ventilation (NIPPV). NIPPV was given to patients for at least 6 to 8 hours, during which time the patients were assessed for clinical stability. Clinical signs and ABGs were checked again after 6 to 8 hours of NIPPV. The presence of sustained clinical improvement with decrease in respiratory rate < 24/minutes, presence of normal pH, PaCO_2_ < 55 mm of Hg and O_2_ saturation more than 90% on ABG was required before patients were considered for weaning from NIPPV. In case of deterioration in patient’s clinical condition and/or ABGs, patients were shifted to Intensive Care Unit (ICU) and followed for a week in terms of requirement for mechanical ventilation and any hospital mortality following intervention failure was recorded.

### Statistical analysis:

Data was analyzed on SPSS Version 10. Frequency and Percentages were computed for qualitative variables like gender, mortality and intubation. Mean and Standard Deviation were computed for quantitative variables like age, respiratory rate, pH, pO_2_ and pCO_2_ before and after NIPPV. Effect modifiers like age and gender were controlled by stratification. Post stratification paired t-test was applied to see difference in the pre- and post- NIPPV quantitative variables. chi-square test was used for qualitative variables. A p-value ≤ 0.05 was considered as significant.

## RESULTS

**Fig. 1:**
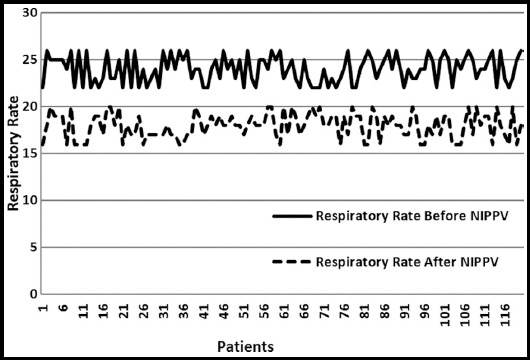
Trends in Respiratory Rate before and after NIPPV.

Out of a total of 120 patients with acute exacerbation of chronic obstructive airway disease, males were 88 (73.3%) and females were 32 (26.7%). Patients age ranged from a minimum of 40 years to a maximum of 75 years with a mean age of 58.88 ± 10.09 (p=0.002). Descriptive statistics for respiratory rate, pH and pCO_2_ are given in Table-I below. The parameters of respiratory rate, pH and carbon dioxide (pCO_2_) were measured before and after NIPPV. The mean respiratory rate of the patients was 24 ± 1.45 per minute before and 17.96 ± 1.35 per minute after NIPPV. This difference was statistically significant with p value of less than < 0.00001. The individual variation in respiratory rate are shown in Fig. 1.

**Table I:** Effect of NIPPV on ABGs and Respiratory Rate (n=120).

	Minimum	Maximum	Mean ± Std. Deviation
Before NIPPV pH	7.20	7.34	7.2703 ± 0.04103
After NIPPV pH	7.35	7.42	7.3851 ± 0.02264
Before NIPPV pCO_2_	45.00	80.00	61.8750 ± 9.60004
After NIPPV pCO_2_	45.00	68.00	57.4667 ± 6.79092
Before NIPPV Resp. Rate	22.00	26.00	24.0083 ± 1.45807
After NIPPV Resp. Rate	16.00	20.00	17.9667 ± 1.35927

**Fig. 2:**
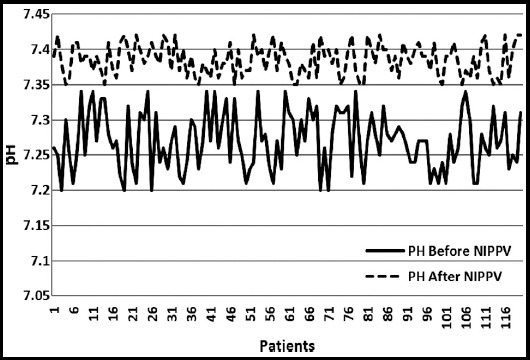
Trends in pH before and after NIPPV.

The mean pH before NIPPV was 7.27 ± 0.04 and afterwards was 7.38 ± 0.02 with statistically significant difference (p < 0.00001) as shown in Fig. 2. The variations in the pH level of the study population before and after NIPPV are highlighted in the figure. The mean pCO_2_ was 61.87 ± 9.60 mm of Hg before and 57.46 ± 6.79 mm of Hg after NIPPV. This difference in pCO_2_ levels was also statistically significant (P < 0.0003). Overall NIPPV was successful in 80% of the patients where the patients were weaned off from NIPPV and discharged home safely.

In our study, twenty four (20%) patients required endotracheal intubation and invasive ventilation compared to 96 (80%) patients who were safely weaned off which was statistically significant (p = 0.011). Out of the 24 patients who were intubated, only five survived making total mortality in hospital as 15.83% (19 out of 120 patients).

## DISCUSSION

We studied the effect of NIPPV on respiratory rate, arterial blood gases, the number of patients who could be discharged home safely, the need for mechanical ventilation and mortality rate in patients with acute exacerbation of COPD. There was significant difference in term of improvement in respiratory rate and ABGs from the baseline. Our results coincide with earlier studies which reported similar findings for respiratory rate and ABGs.^11^,^12^ The beneficial effect of NIPPV in our COPD patients with an acute exacerbation could be due to the fact that non-invasive ventilation reduces respiratory muscle activity and improves the breathing pattern (respiratory rate and tidal volume). In addition, gas exchange improves with increasing pH, PaO_2_ and decreasing PaCO_2_ and reduction in the diaphragmatic activity.^13^,^14^

In our study, 96(80%) patients were discharged home safely while 24 (20%) required mechanical ventilation. All of the 24 patients who did not improve with NIPPV were given mechanical ventilation out of which only 6 (4.2%) recovered. This requires further research into the management of patients who don’t improve with NIPPV. Better hospital survival, fewer intubations, less complications and a shorter hospital stay was found in some studies.^15^,^16^ Nevertheless this needs to be reevaluated in our population as well. Our study results are better than in Bangladesh where NIPPV significantly reduced the need for mechanical ventilation in the non-invasive ventilation group (40%) as compared to 73.3% in the standard group (p=0.01). The in-hospital mortality rate was significantly reduced (16.7%) in the non-invasive group as compared with 43.3% in the standard treatment group (p=0.04).^17^ The mortality in the non-invasive group was comparable to the mortality in our group of patients where it was 15.83%.

While the findings of our study were comparable to the previous studies 5,9,10 in Pakistan in terms of improvement in blood gases and respiratory rate, there was a huge difference in the number of patients requiring mechanical ventilation (20% in our study compared to 5.6% in previous study). 9 The overall mortality in our patients was more (15.83%) compared to previous data (11%).^9^ This difference could be due to the difference in sample size which was very small in the previous study. The high figures with regards to need for mechanical ventilation and mortality warrants more in-depth research into its etiology. Our results concurs with the findings in other parts of the world. This include a systematic review of multiple randomized control trials found that NIPPV improved pH in one hour, PaCO_2_ and respiratory rate and significantly reduced the length of hospital stay^18^ however these studies had a control group for comparison compared to our study. While we did not have a control group, we did compare it with the limited results available in Pakistan. Our results highlighted the overall frequency/need for endotracheal intubation and mortality in patients with acute exacerbation of COPD in our population. This study also provides a baseline for future research into the predictive factors determining morbidity and mortality in such patients.

### Limitations of the study

This was a hospital based cross-sectional study. Large scale hospital-based studies are required as the prevalence of COPD is gradually increasing in Pakistan. While we didn’t use a standard control group, we did compare our results to the data available in Pakistan. Long term follow-up was not done in these patients which could have better explained the outcome. Effect of comorbid conditions was not analyzed which could have contributed to mortality.

## CONCLUSION

There was significant improvement in biochemical and clinical parameters after non-invasive positive pressure ventilation compared to baseline. However, the number of patients with acute exacerbation of COPD in our study requiring mechanical ventilation and the high mortality rate shows that it was underreported from Pakistan previously necessitating the need for further research in our country.

## Author`s Contribution:

**NI:** Conceptualization of study, study design, data collection, analysis and write up.

**NG:** Study design, Literature review, data analysis and write up of manuscript.

**NZ:** Study design, data collection and review of the manuscript.
